# CpG ODN/Mangiferin Dual Delivery through Calcium Alginate Hydrogels Inhibits Immune-Mediated Osteoclastogenesis and Promotes Alveolar Bone Regeneration in Mice

**DOI:** 10.3390/biology12070976

**Published:** 2023-07-10

**Authors:** Yingzhi Gu, Yang Hu, Shengyuan Huang, Sunniva Ruiz, Toshihisa Kawai, Yuxing Bai, Xiaozhe Han

**Affiliations:** 1 Department of Immunology and Infectious Diseases, The Forsyth Institute, 245 First Street, Cambridge, MA 02142, USA; 2 Department of Orthodontics, Beijing Stomatological Hospital, Capital Medical University, Beijing 100050, China; 3 Department of Oral Science and Translational Research, College of Dental Medicine, Nova Southeastern University, Fort Lauderdale, FL 33314, USA; 4 Department of Stomatology, Beijing Tongren Hospital, Capital Medical University, Beijing 100730, China

**Keywords:** alginate hydrogel, CpG ODN, mangiferin, alveolar bone, regeneration, inflammation, osteoclastogenesis

## Abstract

**Simple Summary:**

The immune system plays an important role in the skeletal system during bone repair and regeneration, especially in the oral environment with its constant host–microbe interactions. This study aims to determine the effect of the controlled delivery of immunomodulatory biologicals on alveolar bone regeneration. This study aims to explore the regulatory effect of a hydrogel microbead, coated with multiple immunomodulatory substances (cytosine-phosphate-guanosine oligodeoxynucleotides and glucosylxanthone Mangiferin), on alveolar bone regeneration. The results showed that the complex microbeads could promote the anti-inflammatory response in splenocytes, inhibit RANKL-mediated osteoclastogenesis in a co-culture model of activated splenocytes and osteoclast precursors cells, and promote new bone deposition in mice. This study demonstrated that alveolar bone regeneration could be regulated by cytosine-phosphate-guanosine oligodeoxynucleotides and glucosylxanthone Mangiferin, which provides a potential therapeutic strategy for the treatment of alveolar bone regeneration.

**Abstract:**

The immune system plays an important role in the skeletal system during bone repair and regeneration. The controlled release of biological factors from the immune system could facilitate and optimize the bone remodeling process through the regulation of the activities of bone cells. This study aimed to determine the effect of the controlled delivery of immunomodulatory biologicals on bone regeneration. Immunostimulatory cytosine-phosphate-guanosine oligodeoxynucleotides (CpG ODN) and glucosylxanthone Mangiferin (MAG)-embedded microbeads were incubated with *P. gingivalis*-challenged splenocytes, or co-cultured with RAW264.7 cells. The effect of CpG ODN/MAG-containing microbeads on bone regeneration was then tested in vivo in a mouse alveolar bone defect model. The results demonstrated that MAG significantly antagonized *P. gingivalis* proliferation and reduced the live/dead cell ratio. After the addition of CpG ODN + MAG microbeads, anti-inflammatory cytokines IL-10 and IL-4 were upregulated on day 2 but not day 4, whereas pro-inflammatory cytokine IL-1β responses showed no difference at both timepoints. RANKL production by splenocytes and TRAP+ cell formation of RAW264.7 cells were inhibited by the addition of CpG ODN + MAG microbeads. Alveolar bony defects, filled with CpG ODN + MAG microbeads, showed significantly increased new bone after 4 weeks. In summary, this study evaluated a new hydrogel-based regimen for the local delivery and controlled release of biologicals to repair and regenerate alveolar bony defects. The combined CpG ODN + MAG treatment may promote alveolar bone regeneration through the anti-microbial/anti-inflammatory effects and the inhibition of RANKL-mediated osteoclastogenesis.

## 1. Introduction

The most promising cure for damaged tissue is self-repair and regeneration. Bone tissue can heal without forming scars partially due to its high regenerative capacity [1]. The homeostasis of bone metabolism is maintained by coordinated actions of osteoblasts and osteoclasts. Osteoclasts originate from hematopoietic stem cells (HSCS) and are responsible for bone resorption [2]. They degrade bone by secreting acids and proteolytic enzymes such as Cathepsin K (CTSK), which dissolves collagen and other matrix proteins during bone resorption [3]. Osteoblasts are bone-forming cells, which are generated by the differentiation of mesenchymal precursors into bone progenitor cell lineages through the continuous action of transcription factors, and eventually differentiate into bone cells and are responsible for new bone formation [4]. Under physiological conditions, absorption and formation are stable. However, when the balance is disturbed, bone metabolic diseases such as osteoporosis will occur [5]. This dynamic process allows bones to be continuously reshaped during physiological bone development and wound repair.

There is increasing evidence that effective endogenous bone regeneration requires the coordination of the immune system [6]. The link between the immune and skeletal systems has been established through comprehensive studies in the field of osteoimmunology. It has been found that various factors in the immune system can regulate the activities of osteoblasts and osteoclasts. The controlled release of signals and factors from the immune system is essential to optimize bone remodeling [7]. Generally, pro-inflammatory cytokines, including TNF-α, IL-1, IL-6, and IL-17, enhance osteoclast differentiation, promote bone resorption, and inhibit osteoblast differentiation, function, collagen synthesis, and bone formation [8,9]. On the other hand, the anti-inflammatory cytokines IL-10 and IL-13 show opposite effects that promote osteoblast activity and bone deposition [10,11].

Many drugs have been suggested to promote the repair and regeneration of alveolar bone defects [12,13,14,15]. Immunostimulatory cytosine-phosphate-guanosine (CpG)-containing oligodeoxynucleotides (ODN) is a short single-strand DNA that is recognized by Toll-like receptor 9 (TLR9). CpG ODN has been used as an effective vaccine adjuvant to treat cancer, infectious, and allergic diseases [16,17,18]. Studies have shown that CpG ODN not only co-stimulates CD4^+^ T cells in mice and humans directly but also stimulates B lymphocytes through TLR9-independent mechanisms [19,20]. Studies have shown that local injection of CpG ODN can reduce ligature-induced inflammation and bone loss in experimental periodontitis in mice [21]. Mangiferin (MAG) is a glucosylxanthone isolated from mango tree leaves and mango fruit, which has anti-inflammatory, antibacterial, and antioxidant properties [22]. It enhances the capacity of the monocyte–macrophage system and protects against different human cancers [23]. Furthermore, mangiferin may inhibit osteoclastic bone resorption by suppressing the differentiation of osteoclasts [24].

Polymer-based hydrogel sustained-release systems can improve the bioavailability of biologics by providing localized drug release [25,26]. In the development of many drug delivery dosage forms, natural polysaccharides, such as sodium alginate (SA), are used as biopolymers due to their biocompatibility, biodegradability, high swelling ability, and stability at various pH values [27,28]. Alginate lyase can promote the degradation reaction of alginate gel, effectively solve the disadvantage of slow alginate degradation [29], and can be used to control the rate of gel dissolution and the release of embedded drugs [30,31].

Achieving local controlled drug release by embedding biologicals with alginate hydrogel microbeads can avoid the uncertainty and inconvenience caused by systemic injection of CpG ODN. This allows us to modulate the local immune responses and osteoclastogenesis through the administration of biologicals while minimizing systemic side effects. This study aimed to determine the immune cellular responses to CpG ODN/MAG-containing microbeads, and the effect of these microbeads on bone regeneration in a mouse alveolar bone defect model.

## 2. Materials and Methods

### 2.1. Fabrication of Controlled Degradable Microbeads

Calcium alginate hydrogel microbeads are highly biocompatible materials and are often used as materials for cell encapsulation [32,33]. In order to control the degradation rate of alginate beads, we prepared alginate beads containing alginate lyase with final concentrations of 30 mU/mL, 50 mU/mL, and 70 mU/mL. Calcium alginate microbeads were prepared by adding a cross-linking agent containing calcium ions to realize gelation. Briefly, sodium alginate (Sigma–Aldrich, Darmstadt, Germany) aqueous solution, at a concentration of 1.5% (*w*/*w*), was mixed with alginate lyase (the final concentrations after addition were 30 mU/mL, 50 mU/mL, 70 mU/mL) (Sigma–Aldrich, Darmstadt, Germany). The sodium alginate solution was manually added dropwise to the CaCl_2_ solution (100 mM) through a 27-gauge needle. Gentle agitation of the hardening bath was continued at room temperature until the microbeads gradually solidified. When calcium chloride was added to sodium alginate aqueous solution, the bivalent cation Ca^2+^ and the anion in sodium alginate combined to form a cross-linking point, which led to the cross-linking between molecules and the formation of a three-dimensional network structure. This cross-linked structure gives it a strong ability to maintain its form in water. After 15 min, the microbeads were collected, and the excess calcium ions was eliminated by washing them with deionized water [34].

Images of the beads were taken with a digital camera (3 × fixed zoom) (3.2 Mpix PowerShotA70, Canon, Tokyo, Japan) attached to a binocular microscope (7 × magnification) (Unitron MS, Unitron, Commack, NY, USA) and then analyzed for size and shape by ImageJ software of version 1.53 [35].

### 2.2. Hydrogel Microbeads Encapsulated with CpG ODN/MAG

Four groups of beads were prepared, respectively: (1) base: only 1.5% of sodium alginate solution with 70 mU/mL alginate lyase in the alginate solution; (2) base + CpG ODN; and (3) base + MAG; 4. base + CpG ODN + MAG. Briefly, the 1.5% (*w*/*w*) aqueous solution of sodium alginate was mixed with alginate lyase to make the final concentration of 70 mU/mL. The CpG ODN used in this study was CpG-B K-type (ODN 2006, Hycult Biotech, Wayne, PA, USA) which is a strong B cell activator, Sequence: 5′-tcgtcgttttgtcgttttgtcgtt-3′. CpG ODN and/or Mangiferin (MAG) (Sigma–Aldrich, Darmstadt, Germany) were added to the mixed solution, then propelled dropwise to 100 mM CaCl_2_ solution to prepare the beads 0.7 mm in diameter. CpG ODN was added to make the final concentration of 1 μM, and MAG was added to make the final concentration of 1000 μM in sodium alginate solution.

### 2.3. Bacterial Culture

*P. gingivalis* 33277 (ATCC, Manassas, VA, USA) was grown in either Wilkins-Chalgren broth (Oxoid, Basingstoke, Hampshire, United Kingdom) or on Wilkins-Chalgren agar plates, and incubated in an anaerobic chamber (Coy Laboratory Products, Grass Lake, MI, USA) at 37 °C with 5% CO_2_, 10% H_2_, and 85% N_2_. Frozen stocks of bacterial strains were recovered and were sub-cultured three times through a growth medium before being used in the experiments. The single colony of *P. gingivalis* was isolated from the agar plate and was grown at ATCC bacterial culture medium 2722 (30 g/L tryptic soy broth, 5 g/L yeast extract, 0.5 g/L L-cysteine hydrochloride, 5 mg/L Hemin, 1 mg/L Vitamin K1). After 4 days, the number of bacteria in the culture was determined by the OD readings using a spectrometer and were compared to a standard curve. Bacteria in the logarithmic growth phase were used in all experiments. For the cell culture experiments, bacteria were fixed with 4% paraformaldehyde (PFA) for 30 min at room temperature. Fixed bacteria were then washed three times with sterile PBS and re-suspended in PBS at a concentration of 5 × 10^8^/mL.

### 2.4. Bacterial Live/Dead Cell Detection

To determine the concentration of MAG, four kinds of microbeads with final MAG concentrations of 0 μM, 10 μM, 100 μM, and 1000 μM were fabricated. Prior to *P. gingivalis* inoculation, bacteria were washed three times with PBS and re-suspended in fresh WC anaerobic broth. Bacterial cell counts were determined using a spectrophotometer, where the optical density of 1.0 at 600 nm corresponded to 1 × 10^9^ CFU/mL [36]. The microbeads were placed in a 96-well plate, one per well. MAG microbeads were incubated with 3 × 10^4^ CFU/mL and 3 × 10^6^ CFU/mL density of *P. gingivalis* in bacterial broth for 24 h. After 24 h of incubation, *P. gingivalis* colonization on the surface of the microbeads was detected. Microbeads were washed with PBS to remove the non-adherent bacteria. For the detection of bacteria viability, a live/dead bacterial kit (Molecular Probes, Eugene, OR, USA) was used according to the manufacturer’s instructions. Live bacteria were SYTO 9-positive (green fluorescence), whereas bacteria with compromised membranes were propidium-iodide-positive (red fluorescence). Fluorescent labeling was detected by an inverted epifluorescence microscope (TE2000-S, Nikon, Tokyo, Japan).

### 2.5. Splenocytes Cell Isolation and Culture

Co-culture of *P. gingivalis*-stimulated splenocytes with microbeads was performed as follows: (1) blank: *P. gingivalis*-stimulated splenocytes only; (2) base: *P. gingivalis*-stimulated splenocytes with blank microbeads; (3) base + CpG ODN: *P. gingivalis*-stimulated splenocytes with CpG ODN microbeads; (4) base + MAG: *P. gingivalis*-stimulated splenocytes with MAG microbeads; and (5) base + CpG ODN + MAG: *P. gingivalis*-stimulated splenocytes with CpG ODN + MAG microbeads. For the splenocytes isolation, C57BL/6 mice (8- to 10-week-old males) were euthanized in a CO_2_ chamber. The spleen was aseptically removed from individual mice. Isolated splenocytes were cultured in 96-well plates (200 µL/well) in Iscove’s modified Dulbecco’s medium (IMDM) containing 10% fetal bovine serum (FBS) (Gibico, Waltham, MA, USA), 2 mM L-glutamine, 100 U/mL penicillin, 100 mg/mL streptomycin, 2.5 µg/mL amphotericin B, and 50 µM 2-mercaptoethanol (2-ME) (HyClone, Logan, UT, USA). Splenocytes were sub-cultured into the 96-well plates at 5 × 10^3^ cells/well in the presence of 1 × 10^8^ fixed *P. gingivalis*. After two days, the cells were washed with medium and replated into the 96-well plates. The microbeads were added to the well and incubated for 4 days. The microbeads were placed in a 96-well plate, one per well.

### 2.6. Co-Culture of Splenocytes/RAW264.7 Cells

Co-culture experiments were divided into the following 5 groups: (1) blank: splenocytes/RAW264.7 cells only; (2) base: splenocytes/RAW264.7 cells with blank microbeads; (3) base + CpG ODN: splenocytes/RAW264.7 cells with CpG ODN microbeads; (4) base + MAG: splenocytes/RAW264.7 cells with MAG microbeads; and (5) base + CpG ODN + MAG: splenocytes/RAW264.7 with CpG ODN + MAG microbeads. RAW264.7 cells (ATCC, Manassas, VA, USA) were cultured in Dulbecco’s modified Eagle’s medium (DMEM, HyClone, Logan, UT, USA) containing 10% FBS and 1% penicillin-streptomycin (HyClone, Logan, UT, USA) and incubated at 37 °C with 5% CO_2_. To support osteoclast differentiation, RANKL (50 ng/mL) was added to the culture medium for 2 days [37]. The *P. gingivalis*-stimulated splenocytes (5 × 10^3^ cells/well) and RAW264.7 cells (5 × 10^4^ cells/well) were co-cultured in a 48-well plate. Three microbeads were added to each well and incubated for 4 days. All wells were washed with PBS to remove non-adherent cells followed by tartrate-resistant acid phosphatase (TRAP) staining after co-culture for 4 days.

### 2.7. qPCR

Total RNA from cultured cells and tissues was isolated using a PureLink RNA Mini kit (Life Technologies, Carlsbad, CA, USA) according to the manufacturer’s instructions. The concentration of extracted RNA was measured using a Nanodrop spectrophotometer. RNA samples that had an A260:A280 ratio between 1.8 and 2.2 could be used for further experiments. Each 100 ng mRNA was reverse transcribed into cDNA using the SuperScript II reverse transcriptase in the presence of random primers (Invitrogen, Waltham, MA, USA). qPCR was carried out in a 20 µL reaction mix using Platinum SYBR green quantitative PCR kit (Life Technology, Carlsbad, CA, USA) with a Roche LightCycler 480 instrument (Roche Diagnostics, Indianapolis, IN, USA). GAPDH, IL-10, IL-4, OPG, RANKL, TNF-α, and IL-1β primers were used. The cycling condition was set as: 95 °C for 2 min, followed by 40 cycles of 60 °C for 30 s, and 95 °C for 15 s. Results were normalized by the ΔΔCt method presented as fold changes relative to the value for the GAPDH. Three independent repeats per genotype were carried out. The sequences of the primers were shown in Table 1.

### 2.8. Tartrate-Resistant Acid Phosphatase (TRAP) Staining

The cultured cells were fixed with 4% paraformaldehyde and stained with a TRAP staining kit following the manufacturer’s instructions. TRAP-positive cells containing more than three nuclei were identified, and the images were captured by an inverted phase contrast microscope (Leica ZE4 HD, Leica, Wetzlar, Germany). The percentage of multi-nucleated TRAP+ cells in the cell culture was counted.

### 2.9. Induction of Alveolar Bone Defects in Mice

Mice were purchased from the Jackson Laboratory (Bar Harbor, ME, USA) and used for this study (10 weeks of age). All mice used in the study were maintained under specific pathogen-free (SPF) conditions using cages with an air circulation system and maintained at a temperature of 65 to 75 F and a relative humidity of 50 ± 20%. The cycle of light for 14 h and dark for 10 h was maintained. Animals were fed a standard rodent diet. All the proceedings were approved by the Institutional Animal Care and Use Committee (IACUC) of the Forsyth Institute.

Mice were randomly divided into the following 2 groups (n = 5): (1) bone defect with base microbeads; and (2) bone defect with CpG ODN + MAG microbeads. Under general anesthesia, ketamine (100 mg/kg) and xylazine (5 mg/kg) were injected intraperitoneally for tooth extraction, bone defect preparation, and microbead implantation. To induce experimental bone defects in mice, a 0.7 mm × 0.7 mm × 0.7 mm maxillary alveolar bone defect model was used. Briefly, the maxillary first molar was extracted. After 6 weeks of healing, a 0.7 mm × 0.7 mm × 0.7 mm maxillary alveolar bone defect was made at the first molar site using a drilling handpiece and drill (STERILE FG 247-007) (Dentsply Sirona, NC, USA). Microbead samples were placed inside the bone defect area (1 bead in one defect) and remained for 4 weeks. The hydrogel microspheres have a certain elasticity, and the microspheres would not fall off the defect site after being slightly squeezed into the bone defect sites. All the mice were euthanized by CO_2_ inhalation on day 28 post-surgery.

### 2.10. Micro CT

Formalin was used to treat the maxilla of sacrificed mice. The bone regeneration outcomes were evaluated by scanning with a Scanco micro CT 40 equipment (19-µm voxel size, 70 kVp, 114 mA, 381 ms integration time) (Scanco Medical, Bruttisellen, Switzerland) followed by quantitative three-dimensional (3D) analysis using Mimics software v.21.0 (Materialise, Plymouth, MA, USA). The alveolar bone region of interest (ROI) was selected by setting the position of the bone defect as the center of the cube, the upper edge of the cube was flush with the enamel cementum boundary of the second molar, and the lower edge goes deep into the alveolar bone and approximately flush with the apex of the second molar. The distal edge was at a tangent to the mesial surface of the second molar, the mesial edge extended forward along the alveolar ridge, and a 2 mm spacing distance from the distal mesial margin was determined. The designated ROI was analyzed for differences in cancellous bone parameters. The remaining bone volume (mm^3^) and bone volume/tissue volume (%) were used to analyze the level of bone regeneration. All the measurements for bone resorption were performed without prior knowledge of the group designation of the animals, and a second examiner was used to verify the recordings.

### 2.11. Histological Analysis

Four weeks after treatment, the experiment was terminated, and mice were euthanized for tissue collection. Complete maxillae were dissected, and the excess tissues were removed. After fixing in 10% buffered formaldehyde for 24 h, the specimens were decalcified in 10% EDTA at room temperature. Subsequently, the specimens were dehydrated in graded ethanol and embedded in paraffin. Sections of 8 μm thickness were cut in coronal direction with a microtome. Every 15th slide, for a total of 3 slides, was stained with hematoxylin and eosin (HE) for general healing and bone refill assessment. The outline of the defect was identified in each section and the area was measured as mm^2^ using ImageJ software 1.53.

### 2.12. Statistical Analysis

All the data analyses were expressed as means ± SD. Statistical analysis relative to the control group was performed using *t*-test for two-group comparisons using GraphPad Prism 9 (GraphPad, San Diego, CA, USA). Data analysis for multi-group comparisons was performed by using one-way analysis of variance (ANOVA) and a Tukey multiple comparison test with Bonferroni correction. A probability value of *p* < 0.05 was considered statistically significant.

## 3. Results

### 3.1. Determination of the Degradation Rate of CpG ODN/MAG-Containing Microbeads

**Figure 1:**
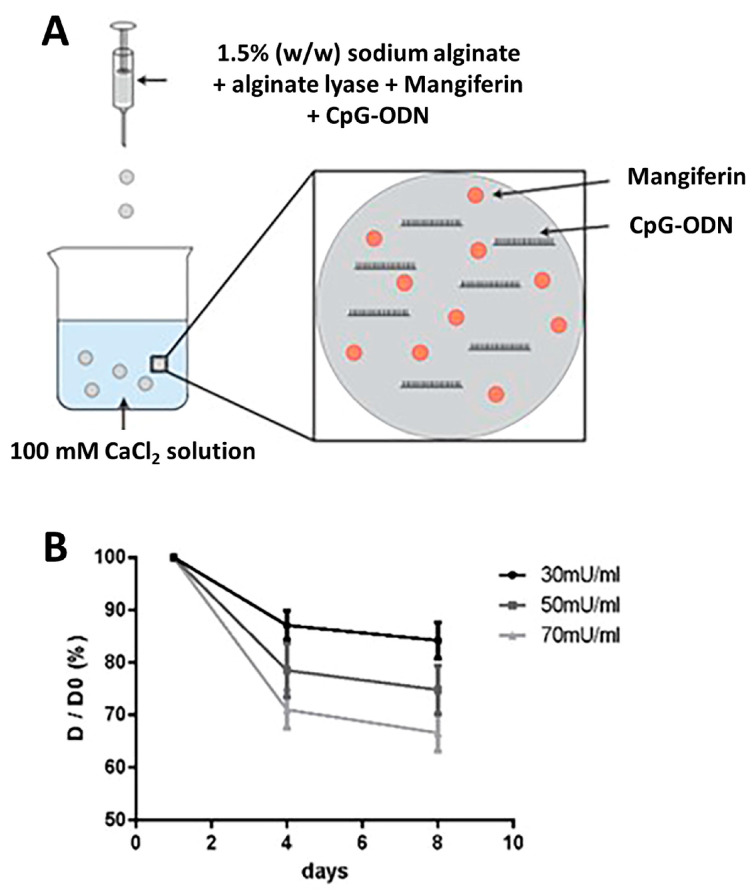
The fabrication of CPG ODN + MAG microbeads and dose-dependent degradation by alginate lyase. (**A**) For the MAG + CPG-embedded hydrogel microbeads preparation, 1 μM CpG ODN and 1000 μM MAG were added to the mixed solution, including a 1.5% (*w*/*w*) aqueous solution of sodium alginate and alginate lyase. The mixture was then introduced dropwise into 100 mM CaCl_2_ solution to form hydrogel microbeads through calcium crosslinking. (**B**) Effect of alginate lyase concentration on bead degradation rate. The diameter of the beads was obtained on Day 0, Day 4, and Day 8 and recorded by a digital camera coupled to a phase contrast light microscope and analyzed by ImageJ software version 15.3t.

To determine the optimal alginate lyase concentration, we soaked alginate beads composed of three alginate lyase concentrations (30 mU/mL, 50 mU/mL, and 70 mU/mL) in the culture medium for 8 days. The diameter of the beads was measured to evaluate the degree of bead dissolution by the alginate lyase digestion. The results showed that the lyase concentration was positively correlated with the reduction in beads diameter. The diameter of the microbeads incubated with 70 mU/mL lyase decreased to 70% of the original diameter after 8 days of incubation, while the diameter of the beads incubated with 30 mU/mL lyase decreased to 90% of the original diameter (Figure 1B). To achieve faster drug release, the lyase concentration of 70 mU/mL was used for subsequent experiments.

### 3.2. Mangiferin Inhibits P. gingivalis Survival

**Figure 2:**
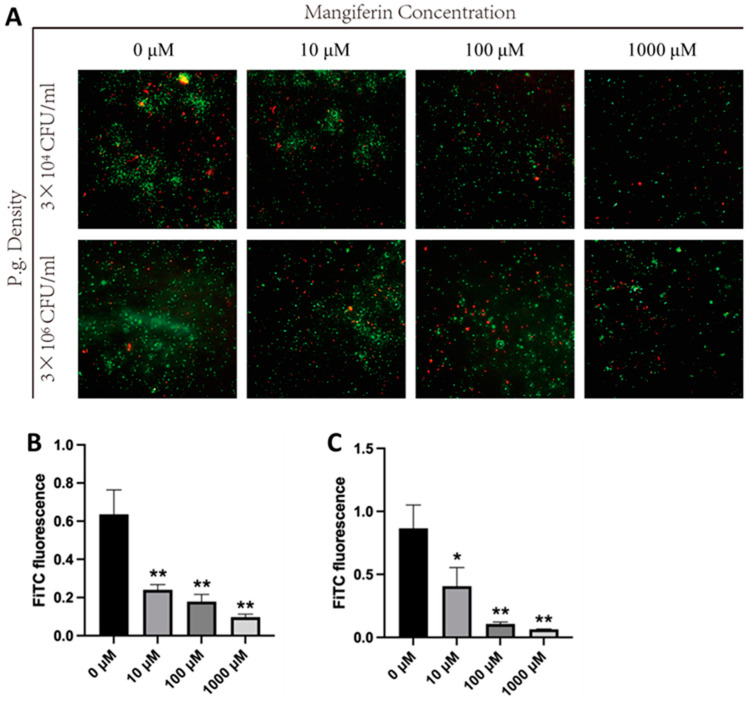
Effect of MAG concentration on the survival of high and low concentrations of *P. gingivalis*. MAG with final concentrations of 0 μM, 10 μM, 100 μM, and 1000 μM was co-cultured with *P. gingivalis* at a density of 3 × 10^4^ CFU/mL and 3 × 10^6^ CFU/mL for 24 h. (**A**) Representative images of live/dead staining of *P. gingivalis* were recorded using a live/dead bacteria kit and a fluorescence microscope. SYTO 9 fluorescent dye was used to stain live bacteria (green), and propidium iodide was used to stain dead bacteria (red). (**B**) Effects of different concentrations of MAG on the survival of *P. gingivalis* at a low concentration (3 × 10^4^ CFU/mL). (**C**) Effects of different concentrations of MAG on the survival of *P. gingivalis* at a high concentration (3 × 10^6^ CFU/mL). ANOVA analysis showed that the inhibition of *P. gingivalis* survival by MAG was dose-dependent. Statistical differences between each experimental group and the 0 μM group were analyzed by *t*-test. The significance (*p*-value) was marked above the bar graph, which was defined as * *p* < 0.05 and ** *p* < 0.01 (n = 3).

The effect of MAG on the survival of *P. gingivalis* was detected by live/dead staining. We tested the effect of different concentrations of MAG on low (3 × 10^4^ CFU/mL) and high (3 × 10^6^ CFU/mL) concentrations of *P. gingivalis*. The results showed that the survival of low concentration of *P. gingivalis* was significantly inhibited by the final concentration of 10 μM, 100 μM, and 1000 μM MAG (Figure 2A,B). The same result was also seen in the high-concentration group (Figure 2A,C). These results suggest that MAG inhibits the survival of *P. gingivalis* in a dose-dependent manner.

### 3.3. CpG ODN + MAG Microbeads Modulated the Expressions of Inflammatory Cytokines and RANKL in P. gingivalis-Challenged Splenocytes

**Figure 3:**
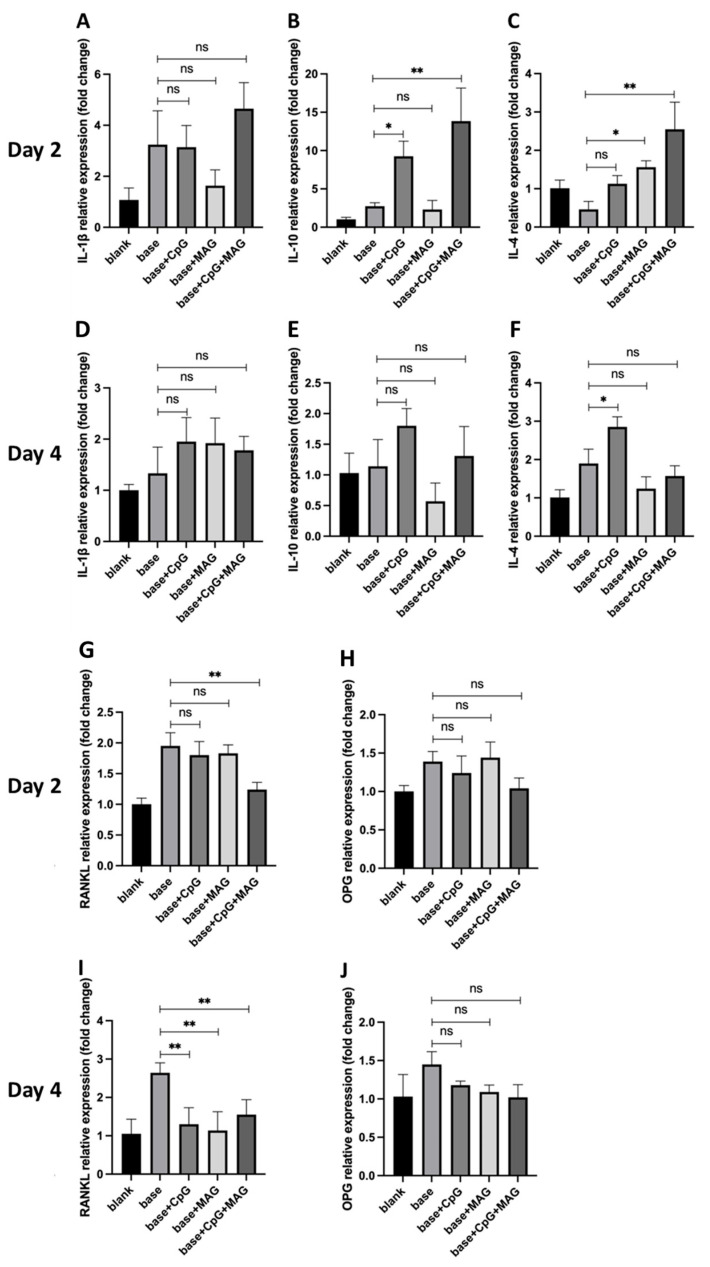
CpG ODN + MAG microbeads induced immune response in splenocytes. The *P. gingivalis*-stimulated splenocytes were co-cultured with base, base + CpG ODN, base + MAG, or base + CpG ODN + MAG microbeads in a 96-well plate for 4 days. (**A**–**J**) The mRNA expression of inflammatory and osteoclast activation-related genes was analyzed on day 2 and day 4 by RT-qPCR (n = 3). The significance (*p*-value) was defined as * *p* < 0.05; ** *p* < 0.01, and no significant difference (ns) *p* > 0.05.

*P. gingivalis*-challenged splenocytes were cultured with microbeads for 2 days or 4 days and the RT-qPCR was performed for the detection of gene transcript levels of pro- and anti-inflammatory cytokines (IL-1β, IL-10, IL-4) and osteoclast modulating factors (RANKL, OPG). No significant changes in IL-1β expression by splenocytes were observed when microbeads were added in each group on both day 2 and day 4 (Figure 3A,D). On day 2, the expression of IL-10 was significantly increased in splenocytes co-cultured with CpG ODN alone or CpG ODN + MAG microbeads, while MAG alone microbeads did not affect the expression of IL-10 (Figure 3B). On day 4, no significant difference in IL-10 expression was observed in splenocytes cultured with CpG ODN alone, MAG alone, or CpG ODN + MAG microbeads, when compared with the base control (Figure 3E). IL-4 expression was significantly increased on day 2 in cells cultured with MAG alone, or CpG ODN + MAG microbeads (Figure 3C), whereas, on day 4, only splenocytes cultured with CpG ODN alone showed a significant increase in IL-4 expression (Figure 3F).

For the osteoclastogenesis-related genes, on day 2, the mRNA expression of RANKL was significantly reduced in splenocytes, only when CpG ODN + MAG microbeads were added in the culture as compared to the control (Figure 3G). On day 4, RANKL expression was significantly reduced in splenocytes when incubated with CpG ODN microbeads, MAG microbeads, or CpG ODN + MAG microbeads (Figure 3I). Osteoprotegerin (OPG) is an inductive receptor for RANKL and it reduces the production of osteoclasts by binding to RANKL. No significant difference in OPG expression was observed in splenocytes incubated with any microbeads for either day 2 or day 4 (Figure 3H,J). These results suggested that CpG ODN and MAG released from microbeads upregulated anti-inflammatory cytokine expression (CpG ODN for IL-10 and IL-4; MAG for IL-4) and down-regulated RANKL expression.

### 3.4. CpG ODN + MAG Microbeads Reduce Osteoclast Activation

**Figure 4:**
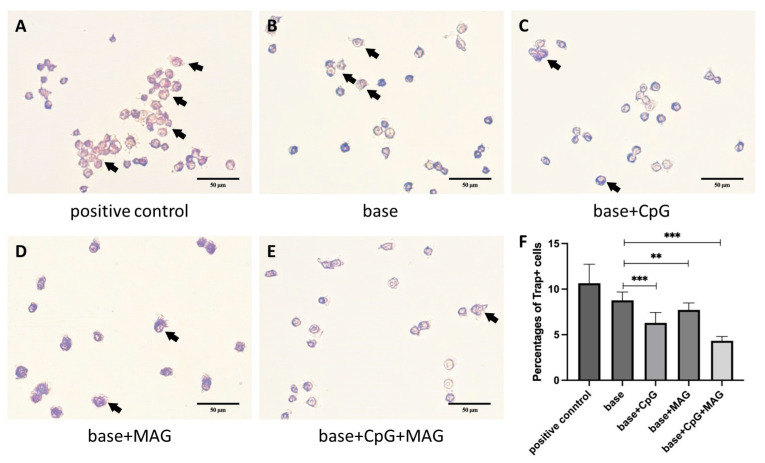
CpG ODN + MAG microbeads inhibited osteoclast activation. The RAW264.7 cells were stimulated with 50 ng/mL RANKL for 2 days, then co-cultured with *P. gingivalis*-stimulated splenocytes (10:1) and incubated with 2 base, base + CpG ODN, base + MAG, or base + CpG ODN + MAG microbeads in a 48-well plate for 4 days. For the positive control, RAW264.7 was co-cultured with splenocytes for 4 days with no microbeads added. (**A**–**E**) The osteoclast activation in different groups were evaluated by TRAP staining. Black arrows indicated the Trap+ cells. (**F**) The percentages of Trap+ cells in different groups (n = 15). The significance (*p*-value) was defined as ** *p* < 0.01, and *** *p* < 0.001.

Since we found that the RANKL expression was reduced by CPG ODN + MAG microbeads in solenocytes, we further investigated whether it could regulate the immune-cell-mediated osteoclastogenesis in RAW264.7 cells. The RAW264.7 cells were stimulated with RANKL and subsequently co-cultured with *P. gingivalis*-stimulated splenocytes. TRAP staining has proceeded to analyze the osteoclast activation. The results showed that the percentage of TRAP+ cells was significantly reduced in base + CpG ODN, base + MAG, and base + CpG ODN + MAG groups compared with the base-only group (Figure 4A–F).

### 3.5. CpG ODN/Mangiferin Microbeads Enhance Alveolar Bone Regeneration in Mice

**Figure 5:**
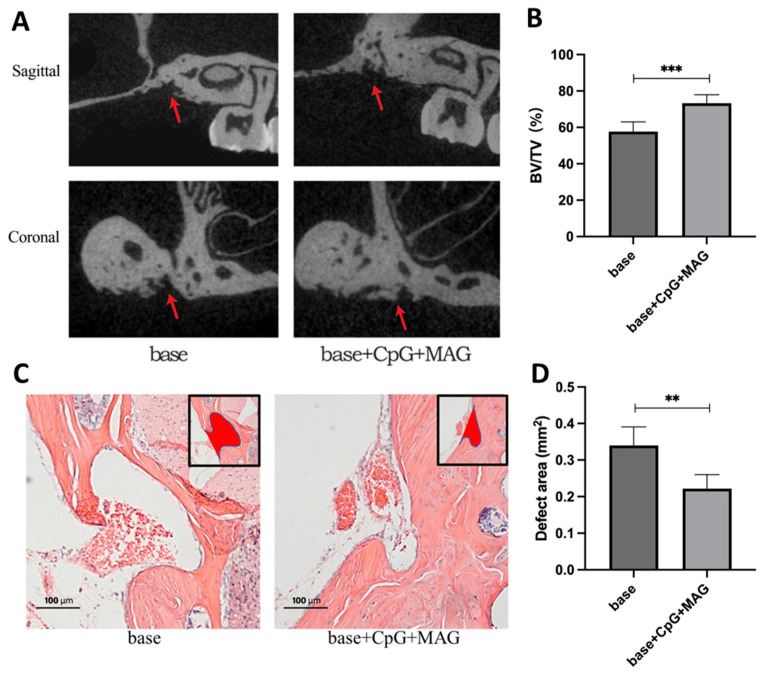
Alveolar bone regeneration induced by CpG ODN + MAG microbeads in mice. A 0.7 mm × 0.7 mm × 0.7 mm maxillary alveolar bony defect was drilled with a 0.7 mm diameter round carbide bur at the extraction site of the first molar. Base microbeads or base + CpG ODN + MAG microbeads were placed inside the bony defect and remained for 4 weeks. (**A**) The representative images of Micro-CT in sagittal and coronal vision. Red arrows showed the bone regeneration of the drilling site. (**B**) The ratio of remaining bone volume to tissue volume (BV/TV) based on Micro-CT was used to analyze the level of bone regeneration (n = 5). (**C**) The representative images of H/E staining. The red areas in the black squares showed the bone defect areas in the different groups. (**D**) Bone defect areas were analyzed between different groups (n = 5). The significance (*p*-value) was defined as ** *p* < 0.01, and *** *p* < 0.001.

Since the in vitro results demonstrated that the microbeads embedded with CpG ODN + MAG were most effective in the inhibition of bacterial growth, in the control of immune inflammatory responses, and in the inhibition of osteoclastogenesis, we tested the bone regenerative induction of this group in vivo using a mouse model of alveolar bone defect. Micro CT results showed that the CpG ODN + MAG beads could significantly increase the ratio of bone volume to tissue volume (BV/TV) (Figure 5A,B), suggesting that CpG ODN + MAG composite material significantly promotes the repair of bone defects compared to the base group. H/E staining showed that the bone defect area of the CpG ODN + MAG group was significantly decreased compared to the base group (Figure 5C,D), further confirming the enhanced bone deposition in the presence of CpG ODN + MAG.

## 4. Discussion

A variety of drug delivery systems, with the advantages of improving drug availability, improving drug stability, and regulating drug release, have been widely reported [38,39,40]. Alginate is a naturally occurring polyanionic polysaccharide extracted from the cell walls of brown algae and certain bacteria [41]. It has been used in a wealth of drug delivery applications due to its excellent biocompatibility and low immunogenicity [42]. Furthermore, due to the susceptibility of glycosidic bonds to enzymatic hydrolysis, alginate is also biodegradable. In addition, alginate can be gelled [43]. Therefore, alginate shows great promise in drug delivery applications. In this study, we constructed an alginate-based CPG ODN and MAG-coated drug delivery system and found that the microbeads exhibit antibacterial, mediate immune regulation, and promote alveolar bone regeneration. The degradation rate of materials is of great significance for guiding tissue regeneration. The spaces formed after bead degradation provide an environment for cell migration and proliferation, blood vessels, and new bone growth [36]. A degradable drug delivery system can be constructed by incorporating alginate lyase into alginate beads, the diameter of which may change over time in an enzyme-dependent manner [44]. Kevin et al. [45] found out that, after a week, hydrogels incorporating 5 and 50 mU/mL of alginate lyase experienced 81% and 91% size reductions. In our experiments, we found that excessively increasing the content of lyase will make the formation of hydrogel microbeads difficult, while reducing the content of lyase will cause the hydrogel microbeads to degrade too slowly. Therefore, we used a lyase dosage of 70 mU/mL. At this lyase concentration, the hydrogel microbeads can shrink to 70% of the original diameter within a week, thereby providing space for the growth of new tissues.

The regulation of the immune system via the manipulation of TLR9 signaling by CpG ODNs is effective in infectious diseases [46], cancers [47], and allergy treatment [48]. Many studies have used microbeads as a medium to dispense CpG ODN [49,50]. Usually, up to 100 μg of free CpG ODN was needed to be administered per mouse to observe biological effects, but with particle encapsulation, effects were observed with the administration of 0.5 μg of CpG ODN. By minimizing the systemic release of CpG ODN, side effects that occur when CpG ODN is given in large amounts can be reduced, which include autoimmunity and lymphoid architectural damage [51]. In our study, microbeads, at a final concentration of 1 μM CpG ODN, can significantly inhibit RANKL expression and inhibit osteoclast differentiation, thus indicating the high drug availability of microbeads.

Our results showed that CpG ODN-containing microbeads can significantly increase the expression of IL-10 and IL-4 mRNA on day 2 and significantly reduce the expression of RANKL on day 4 of co-culture with splenocytes. The K-type CpG ODN used in this study is a strong B cell activator. CpG-ODN activates B cells that produce IL-10, known as B-regulatory cells [52]. The observed IL-10 upregulation in splenocytes may indicate the increase in IL-10-producing B cells, which is a potent immune regulatory component that attenuates bone loss [21,53]. In addition, IL-10-producing B cells may promote bone healing by inhibiting excessive and/or prolonged inflammation [54]. While our results showed no significant changes in pro-inflammatory IL-1β expression after CpG ODN incubation, this does not rule out the possible induction of other pro-inflammatory factors. Indeed, there was a report indicating that K-type CpG ODN, as vaccine adjuvants and immunostimulatory agents, can trigger dendritic cell differentiation and produce proinflammatory cytokines. Structurally distinct classes of synthetic ODN expressing CpG motifs differentially activate immune cells, optimize the sequence and secondary structure of the CpG ODN, and may further improve its immune regulatory function [55]. In addition, it has been reported that the LPS-induced IL-1β expression in bone marrow-derived macrophages could be inhibited by MAG [56]. In this study, we observed a trend of decrease in MAG mRNA expression in splenocytes in base + MAG group, indicating that the MAG may have a potential inhibitory effect on IL-1β expression in immune cells. Alginate hydrogels are biologically inert and are commonly used for biomedical purposes that require minimal inflammation. However, previous studies have shown that Ca^2+^ used in cross-linked calcium alginate gels can have an immunostimulatory effect [57], which may account for the higher expression of RANKL and IL-1β in the base group compared with the blank group.

The co-culture of CpG ODN microbeads and RAW264.7 cells can significantly reduce TRAP+ cell formation. These effects were enhanced by the presence of MAG as cells incubated with CpG ODN + MAG microbeads showed the strongest upregulation of IL-10 and IL-4, down-regulation of RANKL, and reduction in TRAP+ cell formation. Many previous studies have shown that MAG can inhibit inflammatory response [58,59]. Studies have shown that mangiferin can inhibit the bone resorption of osteoclasts by inhibiting osteoclast differentiation and promoting ERβ mRNA expression in mouse bone marrow macrophages, and can significantly reduce the formation of tartrate-resistant acid phosphatase-positive polykaryotic cells [15]. After 8 weeks of periodontitis in mice treated with oral MAG (50 mg/kg body weight, once a day), it was found that MAG significantly inhibited alveolar bone loss, TNF-α production in gingival epithelial cells, and the phosphorylation of NF-κB and JAK1-STAT1/3 pathways [60]. Therefore, MAG has good therapeutic potential in the prevention and treatment of periodontitis. Besides its anti-microbial activity, our results also demonstrated that MAG-containing microbeads inhibited RANKL expression by splenocytes and TRAP+ cell formation by RAW264.7 cells. This is consistent with the reports by others showing that mangiferin positively regulates osteoblast differentiation and suppresses osteoclastogenesis, bone resorption, and RANKL-induced activation of NF-κB and ERK [24,61]. It has been shown that MAG promotes the osteogenic differentiation of stem cells [62,63] and promotes the regeneration of alveolar bone defects [64,65]. Another study showed that CpG ODN increased the early ALP activity of mesenchymal stem cells fourfold without affecting osteoclast formation [66]. Micro-CT and H/E staining results showed that the microspheres containing CpG ODN and MAG promoted bone regeneration at the bone defect site compared with the blank microspheres. This may be related to the regulation of inflammation and the promotion of osteogenic differentiation of stem cells.

The oral environment is unique in its constant exposure to the oral microbiome and host inflammatory responses to microbial changes. In the current study, we used a combination of an immunomodulatory reagent (CpG ODN), and a natural product containing anti-microbial/anti-inflammatory properties (MAG), to cope with the challenge of bone regeneration in the oral cavity. Certain synergistic effects were observed in promoting anti-inflammatory cytokine production and the inhibition of osteoclastogenesis (Figure 3 and Figure 4). In addition, the well-recognized anti-diabetic property of MAG may prove beneficial to facilitate the wound healing and repair of alveolar bony defects in subjects with metabolic abnormalities and disorders [67,68]. Together with the improvement in the locally controlled delivery system, such a bio-compatible regimen may provide novel strategies to achieve optimal alveolar bone repair and regeneration.

It is worth noting that drilling was used to establish a model of alveolar bone defect in mouse experiments, and this method cannot fully simulate the condition of alveolar bone regeneration in the context of the presence of oral bacteria. Nonetheless, this study demonstrated the antibacterial properties of CPG ODN + MAG microbeads in vitro. The positive effect of CPG ODN + MAG microbeads on alveolar bone regeneration is undeniable.

## 5. Conclusions

This study aimed to determine the effect of the controlled delivery of immunomodulatory biologicals on bone regeneration. We synthesized CpG ODN + MAG-loaded hydrogel microbeads and evaluated the controlled release, the antibacterial and immunomodulatory properties, and the effects of biologics on bone regeneration. We found that the combined CpG ODN + MAG treatment may promote alveolar bone regeneration through the anti-microbial/anti-inflammatory effects, and the inhibition of RANKL-mediated osteoclastogenesis. These results suggest that CpG ODN + MAG has the potential for clinical application, which may provide a solution for postoperative alveolar bone regeneration.

## Figures and Tables

**Table 1:** Primers for real-time PCR.

Genes	Forward Primer	Reverse Primer
GAPDH	CCCCAGCAAGGACACTGAGCAA	GTGGGTGCAGCGAACTTTATTGATG
IL-10	ATTTGAATTCCCTGGGTGAGAAG	CACAGGGGAGAAATCGATGACA
IL-4	GGTGCGCCATGCACGGAGATG	TGCGAAGCACCTTGGAAGCCC
OPG	AGCAGGAGTGCAACCGCACC	TTCCAGCTTGCACCACGCCG
RANKL	GGGGGCCGTGCAGAAGGAAC	CTCAGGCTTGCCTCGCTGGG
TNF-α	CAACGCCCTCCTGGCCAACG	TCGGGGCAGCCTTGTCCCTT
IL-1β	ATGCCTTCCCCAGGGCATGT	CTGAGCGACCTGTCTTGGCCG

## Data Availability

The data presented in this study are available on request from the corresponding author.
